# Neck Circumference Is Associated With Hyperuricemia in Women With Polycystic Ovary Syndrome

**DOI:** 10.3389/fendo.2021.712855

**Published:** 2021-09-06

**Authors:** Haiyan Yang, Chang Liu, Congcong Jin, Rong Yu, Lin Ding, Liangshan Mu

**Affiliations:** ^1^Reproductive Medicine Center, The First Affiliated Hospital of Wenzhou Medical University, Wenzhou, China; ^2^Department of Endocrinology and Metabology, The First Affiliated Hospital of Shandong First Medical University, Shandong Provincial Qianfoshan Hospital, Jinan, China; ^3^Department of Reproductive Endocrinology, Women's Hospital and Key Laboratory of Reproductive Genetics (Ministry of Education), Zhejiang University School of Medicine, Hangzhou, China

**Keywords:** uric acid, anthropometric measurement, polycystic ovary syndrome, hyperuricemia, neck circumference

## Abstract

**Objective:**

To evaluate the association between neck circumference (NC) and hyperuricemia in women with polycystic ovary syndrome (PCOS).

**Methods:**

This is a cross-sectional study that recruited 601 women with PCOS from January 2018 to January 2021. PCOS was diagnosed according to the Rotterdam definition. Hyperuricemia was defined as serum uric acid level of at least 357 μmol/L.

**Results:**

PCOS females with hyperuricemia had significantly greater values of NC, body mass index (BMI), waist circumference (WC) and hip circumference (HC). NC was positively associated with serum uric acid levels, with a standardized regression coefficient of 0.34 after adjusting for confounding factors. Furthermore, logistic regression analysis showed that NC was significantly associated with an increased risk of hyperuricemia, with an adjusted odds ratio of 1.36. The associations between NC and serum uric acid levels were more considerable in those with medium/high BMI (BMI ≥ 21.63 kg/m^2^), all ranges of WC or medium/high HC (HC ≥ 90 cm). The optimal cut-off point of NC in predicting hyperuricemia was 32.0 cm (Youden index = 0.48), with the sensitivity and negative predictive value of 84.81% and 92.08%, respectively.

**Conclusions:**

NC was positively correlated with serum uric acid levels and the prevalence of hyperuricemia in women with PCOS. Therefore, we suggest NC as a simple, novel, and reliable anthropometric measure to be used in the routine clinical assessment of women with PCOS to screen those at high risk of hyperuricemia.

## Introduction

Polycystic ovary syndrome (PCOS) is one of the most common endocrine and metabolic disorders in reproductive-aged individuals, with an incidence of 3%-20% according to different diagnostic criteria (1–3). Women with PCOS are characterized by symptoms of hirsutism or oligo/amenorrhea or when resorting to infertility care in early adulthood (4). In addition to reproductive disorders, PCOS is also closely related to a variety of metabolic abnormalities, including insulin resistance, diabetes, dyslipidemia and cardiovascular disease, which exert negative and life-long impacts on quality of life (5).

Uric acid is a metabolite produced during purine metabolism, and elevated serum uric acid levels have been demonstrated as a risk factor of metabolic disorders, including hypertension, diabetes and cardiovascular diseases (6). In addition to the effect on metabolism, recent review suggests that there is a close relationship between serum levels of uric acid and female reproductive disorders (7). Previously, our study demonstrated that the prevalence of hyperuricemia in the PCOS population was almost threefold higher than that in women without PCOS (8). Moreover, serum uric acid can be used as a predictor of adverse pregnancy and foetal outcomes (9, 10). Therefore, the early detection and intervention of hyperuricemia is of great significance for the health of women with PCOS. However, the detection of serum uric acid levels is time-consuming, and it requires professional personnel and specific equipment, which is unapplicable to clinical practice on a large scale, especially in some rural places. In contrast, measuring anthropometric indices such as body mass index (BMI), neck circumference (NC), waist circumference (WC) and hip circumference (HC) using tape or a scale is relatively easy (11). It has been confirmed that BMI (12–14), waist circumference (15–17) and hip circumference are positively correlated with the risk of hyperuricemia in different populations. However, it is not always feasible and accurate to measure WC, HC and BMI in the winter with heavy clothes or postprandially. Therefore, there is a need for a reliable, simple and fast method to identify hyperuricemia early in clinical practice.

Neck circumference (NC) is a convenient anthropometric parameter that reflects the subcutaneous fat tissue of the upper body (18). Our previous study found that in PCOS women with obesity, the prevalence of hyperuricemia was approximately threefold higher than that in PCOS women with normal BMI (8). Therefore, we hypothesized that NC might be a good predictor for hyperuricemia in women with PCOS. It has been reported that NC is a potential predictor of hyperuricemia in the general population (19, 20). However, to the best of our knowledge, there are currently limited studies focused on assessing the association between NC and hyperuricemia in women with PCOS. Thus, we conducted a retrospective study to examine whether NC was associated with serum uric acid levels and the prevalence of hyperuricemia in women with PCOS.

## Patients and Methods

### Participants

This was a cross-sectional study that enrolled 685 PCOS females aged between 20 and 40 years from January 2018 to January 2021 at the reproductive center of the First Affiliated Hospital of Wenzhou Medical University. The exclusion criteria were as follows: 1) patients with other causes of hyperandrogenemia, including congenital adrenal hyperplasia, androgen-secreting neoplasms, and Cushing’s syndrome (n= 4); 2) patients with any medical intervention or diseases that could alter the neck circumference or affect glyco-lipid and uric acid metabolism, including neck surgery (n= 4), neck malformation (n= 2), thyroid dysfunction (n= 8), hypertention (n= 1), tuberculosis (n= 3), malignant tumor (n= 1), and regular oral glucocorticoids (n= 2), oral contraceptives (n= 11) or metformin treatment (n= 6); and 3) patients with incomplete information for anthropometric parameters or laboratory examination (n= 42). Finally, 601 (87.7%) patients were included for further analysis. This study was approved by the Ethics Committee of the First Affiliated Hospital of Wenzhou Medical University. Written informed consent for the whole procedure was obtained from each patient. The study protocol conforms to the ethical guidelines of the Declaration of Helsinki as reflected in the a prior approval by the institution’s human research committee.

### Definitions

Polycystic ovary syndrome was diagnosed according to the Rotterdam definition, in which two of the following three criteria should be met: 1) oligomenorrhoea or amenorrhea (less than eight menstrual cycles in 12 months, or if the menstrual interval was more than 35 days); 2) biochemical or clinical hyperandrogenism (such as hirsutism and acne); and 3) characteristic image of polycystic ovaries (at least one ovary containing 12 or more peripheral follicles measuring 2–9 mm in diameter and/or ovarian volume of at least 10 mL) on transvaginal (frequencies of transducer: 5-7mHz) or abdominal ultrasound (3-5mHz) (21). Hyperuricemia was defined as an SUA level of at least 357 μmol/L (22). Insulin resistance was estimated by the homeostasis model assessment of insulin resistance (HOMA-IR) index as follows: HOMA-IR= fasting blood glucose (FBG, mmol/L) x fasting insulin (FINS, mIU/L)/22.5. The β-Cell function was estimated by the HOMA of β-cell function (HOMA-β) index as follows: HOMA-β= (20×FINS)/(FBG-3.5). The prevalence of hyperuricemia was calculated as the number of patients diagnosed with hyperuricemia divided by the total number of PCOS patients recruited in the study.

### Anthropometric and Laboratory Measurements

The anthropometric measurements included BMI, NC, WC, HC and blood pressure. BMI was calculated as the body weight in kilograms divided by the body height in meters squared (23). Neck circumference was measured using a flexible tape, with the subject remaining standing, head held erect, at the level of the thyroid cartilage (24). WC was measured at the midpoint between the lowest rib and the iliac crest, and HC was measured at the greater trochanter (25). All the measurements of NC, WC and HC were completed by one nurse in our center. Blood pressure was measured with an electronic sphygmomanometer in the sitting position after 10 min of rest. Fasting blood samples were collected after an overnight fast of at least 8 hours during the 2^nd^ to 5^th^ day of the menstrual cycle to measure hormonal and metabolic parameters. All biochemical measurements were tested in the central laboratory of the First Affiliated Hospital of Wenzhou Medical University. Serum luteinizing hormone (LH), follicle stimulating hormone (FSH), estradiol (E2) and testosterone (T) were measured using an autoimmunoassay analyzer [Unicel Dxl 800, Beckman Coulter, USA]. Serum AMH concentrations were analyzed using enzyme-linked immunosorbent assay [DSL,USA]. Fasting plasma glucose, total cholesterol (TC), serum triglycerides (TG), high-density lipoprotein (HDL) and low-density lipoprotein (LDL) were quantified by an autoanalyzer [AU 5800, Beckman, USA].

### Statistical Analysis

Statistical analyses were performed using SPSS version 23.0 software (IBM Corporation), and receiver operating characteristic (ROC) analyses were conducted using MedCalc Application version 19.7.2 software. Data are presented as the median (interquartile range) or as the mean ± standard deviation for continuous variables. Skewness and kurtosis tests for normality were performed and found that the level of basal LH, basal FSH, LH/FSH ratio, basal E2, anti-mullerian hormone (AMH), FINS, HOMA-IR, HOMA-β, TG, and uric acid did not follow normal distributions. Variables with skewed distributions were logarithmically transformed before statistical analysis. Differences between the two groups were analyzed by using Student’s t test for normally distributed continuous variables and the Kruskal-Wallis test for those with skewed distributions. Multivariable linear regression was used to explore the association of NC with serum uric acid level (log-transformed) in different models with adjustment for potential confounders. Binary logistic regression analysis was used to calculate the odds ratios (OR) and 95% confidence interval (CI) of NC for hyperuricemia. For both logistic regression analyses and multivariable linear regression, no variables were adjusted in model 1. Adjusted variables in model 2 included age, SBP, and DBP. In model 3, TG (log-transformed), HDL, FINS (log-transformed), HOMA-IR (log-transformed), HOMA-β (log-transformed) and eGRF (log-transformed) were further adjusted. The interactions of NC with BMI, WC and HC were tested using binary logistic regression. Patients enrolled were stratified into quartiles according to their NC and stratified into low, medium and high groups according to the tertiles of BMI, WC and HC. Receiver operating characteristic (ROC) curves were used to compare the predictive ability of NC, BMI, WC and HC for hyperuricemia by calculating the area under the curve (AUC). The Youden index, defined as sensitivity + specificity – 1, was calculated to identify the optimal cutoff points. The sensitivity and specificity of NC, BMI, WC and HC as well as positive and negative predictive values were calculated for each cutoff point in the sample. All statistical tests were two-sided, and *P*< 0.05 was considered statistically significant.

## Results

### Baseline Characteristics in Women With PCOS

The baseline characteristics categorized by the presence of hyperuricemia in women with PCOS are presented in **Table 1**. The prevalence of hyperuricemia in women with PCOS was 26.29%. Age was matched between the two groups. Compared with women with PCOS without hyperuricemia, women with PCOS with hyperuricemia had significantly greater BMI, NC, WC, HC, SBP, DBP, fasting insulin, HOMA-IR, HOMA-β, TC, TG, LDL, basal T and serum uric acid levels, while the basal FSH and HDL levels were lower (all *P*<0.05). There were no significant differences between the two groups in basal LH levels, E2 levels, AMH, LH/FSH ratio, FBG or eGFR.

**Table 1:** Baseline characteristics of women with PCOS.

Variables	HUA	Non-HUA	*P* value
Number	158	443	–
Age (year)	29.50±3.68	29.52±3.82	0.96
BMI (kg/m^2^)	26.14±3.29	22.65±3.25	<0.001
NC (cm)	34.68±2.46	31.92±2.10	<0.001
WC (cm)	86.06±8.85	77.23±8.92	<0.001
HC (cm)	98.41±7.27	91.76±7.09	<0.001
SBP (mmHg)	118.82±12.47	110.61±11.87	<0.001
DBP (mmHg)	79.17±9.35	73.70±8.50	<0.001
Basal LH (IU/L)	6.64 (4.07-9.74)	6.93 (4.67-10.12)	0.54
Basal FSH (IU/L)	6.67 (5.61-7.51)	6.74 (5.66-8.13)	0.03
LH/FSH ratio	1.03 (0.69-1.51)	1.02 (0.70-1.49)	0.66
Basal E2 (pmol/L)	173.00 (121.00-213.00)	170.00 (116.00-224.00)	0.80
Basal T (nmol/L)	2.22±0.82	2.01±0.79	0.01
AMH (ng/mL)	8.90 (6.21-11.60)	8.16 (6.15-11.08)	0.60
FBG (mmol/L)	5.41±0.91	5.27±1.03	0.14
FINS (mIU/L)	15.97 (12.13-22.86)	9.80 (6.78-13.56)	<0.001
HOMA-IR	3.75 (2.83-5.82)	2.22 (1.54-3.22)	<0.001
HOMA-β	187.06 (121.39-269.47)	120.90 (83.23-167.27)	0.08
TC (mmol/L)	5.14±1.03	4.91±0.91	0.01
TG (mmol/L)	1.67 (1.20-2.29)	1.14 (0.80-1.64)	<0.001
HDL (mmol/L)	1.20±0.24	1.39±0.33	<0.001
LDL (mmol/L)	3.07±0.86	2.80±0.75	<0.001
Uric acid (μmol/L)	410.00 (378.75-446.00)	284.00 (252.00-312.00)	<0.001
eGFR (mL/min/1.73m^2^)	123.62±7.20	125.48±8.76	0.21

Note: Variables are expressed as mean ± standard deviation or median (interquartile range).

Abbreviations: PCOS, polycystic ovary syndrome; HUA, hyperuricemia; BMI, body mass index; NC, neck circumference; WC, waist circumference; HC, hip circumference; SBP, systolic pressure; DBP, diastolic pressure; LH, luteinizing hormone; FSH, follicle stimulating hormone; E2, estradiol; T, testosterone; AMH, anti-mullerian hormone; FBG, fasting blood glucose; FINS, fasting insulin; HOMA-IR, homeostasis model assessment of insulin resistance; HOMA-β, homeostasis model assessment of β cell function; TC, total cholesterol; TG, triglycerides; HDL, high-density lipoprotein; LDL, low-density lipoprotein.

### Associations of NC With Serum Uric Acid and Hyperuricemia

Multivariable linear regression analysis was performed to explore the associations between NC and serum uric acid levels (**Table 2**). In unadjusted model 1, NC was significantly associated with the level of uric acid, and the standardized coefficient was 0.52 (*P*<.001). In model 2 (adjustment for age, SBP, and DBP) and model 3 (further adjustment for BMI, WC, HC, log TG, HDL, log FINS, log FSH and log T), NC was still significantly associated with the level of uric acid, and the standardized coefficients were 0.48 (*P*<0.001) and 0.34 (*P*<0.001), respectively.

**Table 2:** Association of neck circumference with serum uric acid level and hyperuricemia in women with polycystic ovary syndrome.

	Linear regression on Log (serum uric acid level)		Logistic regression on the hyperuricemia		
Variables	Standardized coefficient	*P* value	OR	95% CI	*P* value
Model 1, NC	0.52	<0.001	1.75	1.57-1.95	<0.001
Model 2, NC	0.48	<0.001	1.67	1.49-1.87	<0.001
Model 3, NC	0.34	<0.001	1.36	1.15-1.60	<0.001

Model 1 was unadjusted. Model 2 was adjusted for age, SBP and DBP. Model 3 was further adjusted for BMI, WC, HC, TG (log-transformmed), HDL, FINS (log-transformmed), basal FSH (log-transformmed) and basal T (log-transformmed). N,, neck circumference; SBP, systolic pressure; DBP, diastolic pressure; BMI, body mass index; WC, waist circumference; HC, hip circumference; TG, triglycerides; HDL, high-density lipoprotein; FINS, fasting insulin; FSH, follicle stimulating hormone; T, testosterone; CI, confidence interval; OR, odds ratio.

In addition, binary logistic regression analysis was also conducted to further identify the correlation between NC and hyperuricemia (**Table 2**). In model 1 without any adjustment, the OR (95% CI) was 1.75 (1.57–1.95: *P*<0.001). In model 2 and model 3, with the same adjustment as those in multivariate linear regression analysis, the correlation between NC and hyperuricemia was still statistically significant, and the ORs (95% CI) were 1.67 (1.49–1.87; *P*<0.001) and 1.36 (1.15–1.60; *P*<0.001), respectively.

### Interactions of NC With Other Anthropometric Measurements in Relation to Serum Uric Acid Level

**Figure 1:**
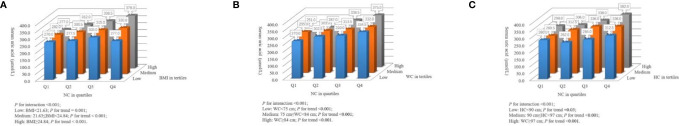
Interactions of NC with other anthropometric measurements in serum uric acid level. **(A)** The joint effect of NC (in quartiles) with BMI (low, medium, and high levels) on serum uric acid level. **(B)** The joint effect of NC (in quartiles) with WC (low, medium, and high levels) on serum uric acid level. **(C)** The joint effect of NC (in quartiles) with HC (low, medium, and high levels) on serum uric acid level. NC, neck circumference; BMI, body mass index; WC, waist circumference; HC, hip circumference.

The quartile ranges of NC were < 31.0 cm (n=203), 31.0 cm to < 32.0 cm (n=100), 32.0 cm to < 34 cm (n=154), and ≥ 34 cm (n=144). The tertile ranges of BMI were < 21.63 kg/m^2^ (n=202), 21.63 kg/m^2^ to < 24.84 kg/m^2^ (n=202), and ≥ 24.84 kg/m^2^ (n=197). The tertile ranges of WC were < 75 cm (n=221), 75 cm to < 84 cm (n=189), and ≥ 84 cm (n=191). The tertile ranges of HC were < 90 cm (n=227), 90 cm to < 97 cm (n=199), and ≥ 97 cm (n=175). There were significant interactions of NC with BMI, WC and HC (*P* for interaction <0.001) in relation to serum uric acid level (**Figure 1**). The associations between NC and serum uric acid levels were more considerable in those with medium/high BMI (BMI ≥ 21.63 kg/m^2^, *P* for trend <0.001), all ranges of WC (*P* for trend <0.001) or medium/high HC (HC ≥ 90 cm, *P* for trend <0.001).

### The Predictive Ability of NC for Hyperuricemia

**Figure 2:**
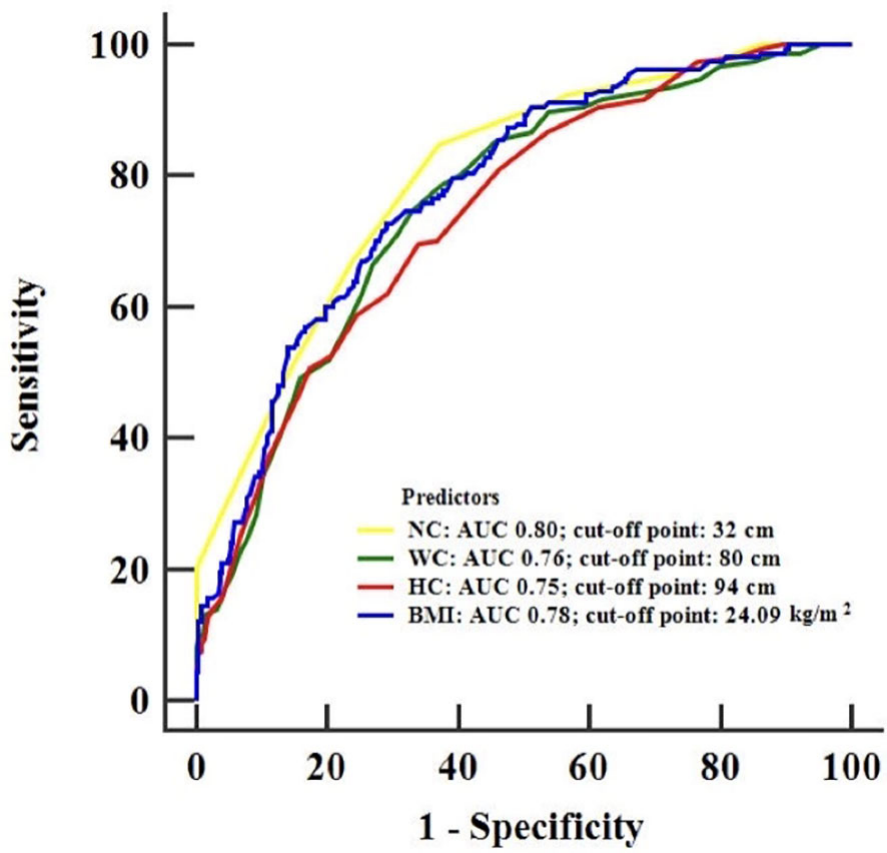
Receiver operating characteristic curves for the detection of hyperuricemia using NC, BMI, WC and HC. NC, neck circumference; BMI, body mass index; WC, waist circumference; HC, hip circumference.

Receiver operating characteristic (ROC) analysis was used to determine the predictive ability of NC, BMI, WC and HC for hyperuricemia. The areas under the curve (AUCs) for NC, BMI, WC and HC in predicting hyperuricemia are depicted in **Figure 2**. The AUC (95% CI) for NC was 0.80 (0.77–0.83), which was significantly larger than that for WC and HC, with AUCs (95% CI) of 0.76 (0.72–0.79) and 0.75 (0.71–0.78), respectively. However, no significant differences were found in the AUCs between NC and BMI (**Supplementary Table 1**). The different cutoff points, sensitivities, specificities, positive and negative predictive values of NC, BMI, WC and HC are shown in **Supplementary Table 2**. The optimal cutoff points of NC, BMI, WC and HC in predicting hyperuricemia were 32.0 cm (Youden index = 0.48), 24.09 kg/m^2^ (Youden index = 0.44), 80.0 cm (Youden index = 0.42) and 94 cm (Youden index = 0.36), respectively. The sensitivity (SE) and negative predictive value (NPV) of NC were 84.81% and 93.68%, which were comparatively higher than those of BMI (SE: 72.78%; NPV: 87.95%), WC (SE: 74.68%; NPV: 88.13%) and HC (SE: 69.62%; NPV: 85.92%).

## Discussion

In the current study, we demonstrated that neck circumference was positively associated with serum uric acid levels and was also significantly correlated with the prevalence of hyperuricemia in women with PCOS. We also observed significant interactions between BMI, WC and HC with NC in relation to serum uric acid concentrations in women with PCOS. Additionally, ROC analysis demonstrated that NC had a higher negative predictive value for hyperuricemia in women with PCOS than BMI, WC, and HC. To the best of our knowledge, this is the first study to demonstrate such a correlation between neck circumference and hyperuricemia in PCOS women.

Hyperuricemia is characterized by an abnormal increase in serum uric acid levels in the human body due to aberrant purine metabolism, abnormal renal secretion and reabsorption (26, 27). Studies have demonstrated that elevated serum uric acid levels are associated with increased risks of hypertension, type 2 diabetes and cardiovascular diseases (28–30), which has received increasing attention as a major public health problem (31). Our previous study showed that the prevalence of hyperuricemia in the PCOS population was 25.48% (8), which is almost threefold higher than that of women in the general population (32). In the present study, the results are in line with our previous findings that showed that 26.29% of PCOS patients were diagnosed with hyperuricemia. Therefore, it is of great importance to find a simple detection method for early recognition of high-risk populations during symptomless periods. Our previous study also found that 58.75% of women with PCOS and obesity had hyperuricemia, which was nearly threefold higher than that in women with PCOS and a normal BMI, which indicated that accumulated fat was positively associated with a high risk of hyperuricemia (8).

As a reliable anthropometric index of upper-body subcutaneous fat, neck circumference has the advantages of convenience and standardized measurement and has been widely applied in the screening of abnormal fat distribution (33). It has been well acknowledged that NC is positively associated with the risk of obstructive sleep apnea (34), insulin resistance (35), type 2 diabetes and metabolic syndrome (36) in the general population. For women with PCOS, studies have shown that NC was positively correlated with visceral fat and could be adopted as an innovative tool for assessing body adiposity distribution (37, 38). Several studies have suggested that body fat accumulated in the upper body segment may contribute to hyperuricemia (12, 39). Each additional 5 mm increase in NC was associated with a 17% higher likelihood of having hyperuricemia in women (20). In the current study, we also found a positive association between NC and the level of uric acid. Further adjustment for age and other confounders still found a positive association between NC and hyperuricemia, suggesting that neck circumference was independently associated with the prevalence of hyperuricemia in PCOS patients. We also observed significant interactions between BMI, WC and HC with NC in relation to serum uric acid concentrations in women with PCOS. Interestingly, the associations between NC and serum uric acid level were more considerable in those with medium/high BMI (BMI ≥ 21.63 kg/m^2^), or medium/high HC (HC ≥ 90 cm) regardless of the waist circumference, which suggests that NC as an indicator for upper-body subcutaneous fat is a pathogenic and independent fat depot that confers additional risks for hyperuricemia. In addition, our results showed that the area under the curve of NC in predicting hyperuricemia appeared significantly larger than that of WC and HC. NC has good sensitivity and negative predictive value for the identification of hyperuricemia, which could avoid unnecessary medical intervention in some ways.

Several potential mechanisms account for the high prevalence of hyperuricemia in women with PCOS and larger NCs. First, recent compelling evidence indicates that in women with PCOS, NC is a good predictor for insulin resistance (IR) (40), which consequently increases the risk of hyperuricemia by reducing the excretion of uric acid through increased proximal tubular sodium reabsorption (26, 41). Second, since an increased prevalence of obstructive sleep apnea has been observed in women with PCOS (42), xanthine oxidase, an enzyme that plays a key role in uric acid synthesis, could be activated under hypoxic conditions in those patients (43). Third, increasing evidence has shown that more than 60% of free fatty acids (FFAs) are released from upper-body subcutaneous adipose tissue (44). High levels of FFAs exert pathogenic effects on the glomerulus (44) and tubulointerstitium (45), leading to abnormal renal secretion and reabsorption of uric acid (27, 46). In summary, the underlying mechanism of higher uric acid levels in PCOS women with larger NCs lies in more free fatty acid release and higher airway pressure from upper body adiposity tissue, both of which could result in oxidative stress and insulin resistance (20).

This study is the first to assess the association between neck circumference and the prevalence of hyperuricemia in women with PCOS. The strengths of our study lie in the simple and standardized measuring method of neck circumference, as well as the validated and complete metabolic data, which make our findings highly applicable to clinical practice. Moreover, the high Youden index, high sensitivity and high negative predictive value of NC in predicting hyperuricemia could avoid unnecessary medical intervention in the clinical practice. However, several limitations should be taken into consideration. First, the single-center retrospective design has some limitations with regard to interpreting the causality of associations. Second, due to limited equipment, the dosage of steroid hormones was not carried out with mass spectrometry but with an autoimmunoassay analyzer. Third, we did not classify the phenotypes of PCOS and did not calculate the prevalence of hyperuricemia in each phenotype. Thus, prospectively designed studies and follow-up of reproductive outcomes await further evaluation.

## Conclusions

In summary, NC was positively correlated with serum uric acid levels and the prevalence of hyperuricemia in women with PCOS. Therefore, neck circumference could be recommended as a comparatively simple, fast, and reliable measuring method in the routine clinical assessment of women with PCOS to screen those at high risk of hyperuricemia.

## Data Availability Statement

The raw data supporting the conclusions of this article will be made available by the authors, without undue reservation.

## Author Contributions

HY and CL drafted and finished the manuscript equally. CJ participated in the collection of data and literature. RY participated in the statistical analysis. LM and LD designed and revised the manuscript. All authors contributed to the article and approved the submitted version.

## Funding

This work was supported by the National Natural Science Foundation of China (82001503), the China Postdoctoral Science Foundation (2021T140600, 2020M671760), and the Wenzhou Municipal Science and Technology Bureau Foundation of Wenzhou, Zhejiang, China (Y2020517).

## Conflict of Interest

The authors declare that the research was conducted in the absence of any commercial or financial relationships that could be construed as a potential conflict of interest.

## Publisher’s Note

All claims expressed in this article are solely those of the authors and do not necessarily represent those of their affiliated organizations, or those of the publisher, the editors and the reviewers. Any product that may be evaluated in this article, or claim that may be made by its manufacturer, is not guaranteed or endorsed by the publisher.
