# Supplementary material for: Neck Circumference Is Associated With Hyperuricemia in Women With Polycystic Ovary Syndrome
Source: Front Endocrinol (Lausanne). 2021 Sep 6;12:712855. doi: 10.3389/fendo.2021.712855 (PMC8450923; doi:10.3389/fendo.2021.712855)
Supplement: Supplementary file 1 [file Table_1.docx]

**Supplementary Table 1. Comparisons of AUC between NC and**

**other anthropometric measurements for hyperuricemia**

|  | *P*-value |
| --- | --- |
| NC vs. BMI | 0.24 |
| NC vs. WC | 0.01 |
| NC vs. HC | 0.003 |

NC = neck circumference; BMI = body mass index; WC = waist circumference

HC = hip circumference; AUC = area under the curve
