# Supplementary material for: Neck Circumference Is Associated With Hyperuricemia in Women With Polycystic Ovary Syndrome
Source: Front Endocrinol (Lausanne). 2021 Sep 6;12:712855. doi: 10.3389/fendo.2021.712855 (PMC8450923; doi:10.3389/fendo.2021.712855)
Supplement: Supplementary file 2 [file Table_2.docx]

**Supplementary Table 2. AUC, cut-off points, sensitivities, specificities, positive and negative predictive values of anthropometric measures for hyperuricemia**

| Variables | AUC | Cut-off points | Youden Index | SE (95% CI) | SP (95% CI) | PPV (%) | NPV (%) |
| --- | --- | --- | --- | --- | --- | --- | --- |
| NC (cm) | 0.80 | 32.00 | 0.48 | 84.81 (78.20 - 90.00) | 62.98 (58.30 - 67.50) | 44.97 | 92.08 |
| BMI (kg/m^2^) | 0.78 | 24.09 | 0.44 | 72.78 (65.10 - 79.60) | 70.88 (66.40 - 75.10) | 47.13 | 87.95 |
| WC (cm) | 0.76 | 80.00 | 0.42 | 74.68 (67.20 - 81.30) | 67.04 (62.40 - 71.40) | 44.69 | 88.13 |
| HC (cm) | 0.75 | 94.00 | 0.36 | 69.62 (61.80 - 76.70) | 66.14 (61.50 - 70.50) | 42.31 | 85.92 |

NC = neck circumference; BMI = body mass index; WC = waist circumference; HC = hip circumference; AUC = area under the curve; 95% CI = 95% confidence interval; SE = sensitivity; SP = specificity; PPV = positive predictive value; NPV = negative predictive value.
